# Influence of Diamond Grain Size on the Basic Properties of WC-Co/Diamond Composites Used in Tools for Wood-Based Materials Machining

**DOI:** 10.3390/ma15103569

**Published:** 2022-05-17

**Authors:** Joanna Wachowicz, Jacek Wilkowski

**Affiliations:** Department of Mechanical Processing of Wood, Institute of Wood Sciences and Furniture, Warsaw University of Life Sciences, Nowoursynowska Street, 166, 02-787 Warsaw, Poland; jacek_wilkowski@sggw.edu.pl

**Keywords:** sintering, cemented carbides WC-Co, powder metallurgy

## Abstract

The paper presents the effect of diamond particle size (varying between 2.5 µm and 20 µm) on the microstructure, density and hardness of WC-Co/diamond composites. The obtained materials contained 30% vol. diamond. The advanced sintering method Pulse Plasma Sintering (PPS) was used for the production of composites. The sintering process was carried out in two stages at a pressure of 50 and 100 MPa and a temperature of 1050 °C. Depending on the size of the diamond particles, composites with a density of 91–99% were obtained. Microstructure studies were performed employing scanning electron microscopy, along with an analysis of the chemical composition in micro-areas. Additionally, the phase composition was investigated by means of X-ray diffraction. In addition, hardness tests were performed. It was found that the size of the diamond particles significantly influenced the microstructure of the tested materials, as well as the density and hardness. As a result of PPS sintering of composites containing the finest diamond particles (2.5–5 µm), the presence of a metastable type of diamond—graphite was found.

## 1. Introduction

Sintered carbides WC-Co are widely used tool materials. These materials are characterized by high hardness and resistance to abrasive wear. Due to the development of the furniture industry, the demand for new tool materials has increased in recent years, providing increasingly higher machining parameters and improved performance. The researchers’ works are focused mainly on improving the cutting and strength properties of WC-Co carbides, by: hard coating, ion implantation or development of new composite materials based on WC-Co [1,2,3,4,5,6,7,8,9,10,11,12,13,14,15]. Despite all efforts of materials engineering, the “ideal tool material” has not yet been developed. A WC-Co composite with dispersed diamond particles could be the material that would ensure appropriate processing parameters and high durability at a relatively low production cost. Carbide matrix would ensure high resistance to abrasive wear and brittle fracture whereas the diamond particles—high hardness.

Producing a WC-Co composite with diamond is not simple. Diamond is a metastable, allotropic variant of carbon and undergoes a transformation into a stable phase that is graphite easily. It is only at a pressure within 3–12 GPa that the stability area for the diamond occurs. The process of graphitization is favored by metals from the VIII group of the periodic table (Co, Ni, Fe). Moreover, this phenomenon increases with the growth of temperature and time of sintering.

The activation energy of the transformation of diamond into graphite also depends on the internal and external crystal structure. The stability of diamond is negatively affected by defects in the diamond crystals in the form of metallic and non-metallic inclusions, originating mainly from the catalyst materials from the diamond synthesis process. The rate of transformation of diamond to graphite also depends on the annealing atmosphere [16].

Studies of the process of diamond graphitization in a high vacuum (low partial pressure of oxygen) indicate that in the temperature range 970–1670 K the transformation of diamond to graphite proceeds slowly on the surface of diamond particles and, at temperatures higher than 2170 K, it proceeds rapidly throughout its volume [17,18,19,20].

The graphitization process of the diamond largely depends on the size of diamond particles. [21] In this paper, the graphitization of nanodiamond (5 nm) in different gaseous atmospheres was studied [22]. It turned out that the temperature of onset of the nanodiamond graphitization in argon is only 940 K, which is much lower than that of a diamond with larger particles, which starts from a temperature above 1800 K in an inert gas under atmospheric pressure. This difference is due to the high surface-to-volume ratio and high thermal conductivity of the diamond [23].

The performance of tools made from diamond composites depends largely on the adhesion of the diamond particles to the matrix. The adhesion connecting the diamond particles to the matrix can be improved by applying a coating on the diamond particles. The coatings play two roles—improve the adhesion of the diamond particles to the matrix and prevent graphitization processes.

In studies [24,25], WC-Co composites with dispersed diamond particles were obtained. To ensure good bonding with the matrix, diamond particles were covered with a layer of tungsten or SiC. No graphite was observed in the composites obtained. On the other hand, the resistance to brittle fracture was twice as high compared to WC-Co carbides. The increase of resistance to fracture toughness is related to the blocking or dispersion of propagating cracks on the diamond particles.

Given the properties of diamond, it is not possible to obtain tungsten carbide composite with dispersed particles of diamond using conventional methods of sintering. Graphitization of diamond can be avoided by sintering at lower pressure (low partial pressure of oxygen) at as low a temperature as possible and in as short a time as possible (up to few minutes). Such conditions of sintering are enabled by a prototype device for PPS (Pulse Plasma Sintering). In this device, the powder is heated using short pulses (of several hundred microseconds) with an amperage of up to several dozen kiloamperes. Such sintering conditions make it possible to obtain dense sinter within a few minutes at a pressing pressure not exceeding 100 MPa.

The mechanism of heating up powder in the prototype PPS method is similar to the mechanism which occurs in the SPS technique. However, the PPS process uses higher impulse amperage as well as higher voltage, shorter duration of current impulse, and longer time intervals between the subsequent current impulses. Powder heating up in the PPS method takes place as a result of cyclic discharging of capacitors. Amperage flowing through the sintered powder at the time of discharging the capacitors reaches the value of several dozen kA and the duration is several hundred microseconds. During the flow of electric impulse, the powder is heated up to a high temperature and, after its disappearance, it undergoes cooling down to the set sintering temperature [26,27,28,29,30,31].

The aim of this study was to obtain a WC-Co composite with dispersed diamond particles with a density close to the theoretical one and to determine the influence of diamond particle size on the hardness and density of the produced composites.

## 2. Materials and Methods

The density of the obtained sinters was measured with the Archimedes method with the use of the Gibertini E154 scale with the equipment for solids density measurement. The phase composition of the obtained sinters was determined by means of Philips PW 1140 diffractometer (Philips, Amsterdam, The Netherlands) with PW 1050 goniometer using Co Kα radiation. Micro-structure of the metallo-graphic samples and fractures was observed by means of scanning electron microscope Hitachi S3500N (Hitachi, Tokyo, Japan).

Hardness measurements were carried out using the Vickers (London, UK) using a FutureTech FM-700 hardness tester (Tokyo, Japan) with a load of 9.807 N.

**Figure 1:**
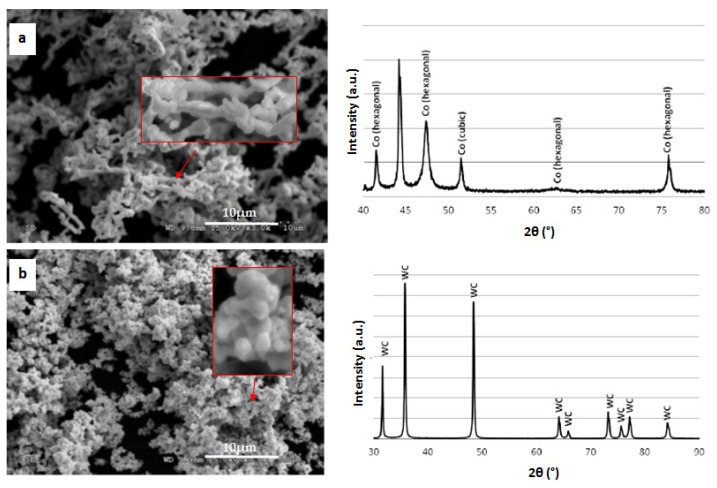
SEM image and XRD analysis: (**a**) cobalt powder, (**b**) WC powder.

Ultra-fine (<100 nm in size) cobalt powder, WC, and diamond were used to produce the sinter. SEM images and XRD analysis of cobalt powder and WC are presented in Figure 1. Cobalt particles are characterized by an irregular, elongated yet rounded shape. The WC powder used is also characterized by an irregular shape and the powder particles form agglomerates of different sizes.

**Figure 2:**
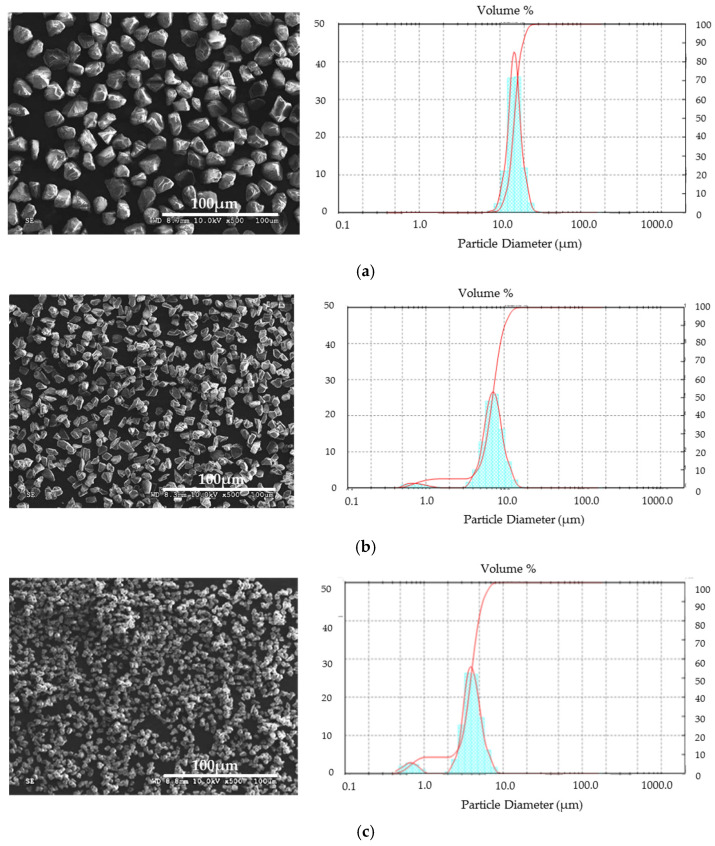
SEM image and particle size distribution of starting diamond powders. (**a**) 16–20 μm; (**b**) 8–10 μm; (**c**) 2.5–5 μm.

Diamond, manufactured by Luoyang Qiming Superhard Material Co., Ltd. (Luoyang, China), with the following gradations (designated by the manufacturer), was used for the study: 2.5–5 μm, 8–10 μm, and 16–20 μm. SEM images of individual diamond powders are presented in Figure 2. Verification of the diamond grain size was carried out with the use of laser diffraction. A Mastersizer particle size analyzer (Malvern Panalytical, Malvern, UK) was used for the tests. Figure 2 presents test results, which confirmed the size range given by the manufacturer. It is worth noting that powders with smaller diamond diameters: 8–10 μm and 2.5–5 μm, had a higher tendency to form agglomerates. Therefore, the particle size distribution assumes a bimodal character.

Mixing is one of the basic elements in the technological process of powder metallurgy. The final quality of the material microstructure, which directly affects its properties, depends on good mixing of the individual components. For the mixing of powders, small balls are often used to further assist the process. Small balls are used when mixing hard and brittle powders, in particular. They make it easier for the more plastic component to cover the much harder grains in the mixture. Powder mixtures, from which sintering is made, are prepared in a turbula-type mixer. Carbide balls with diameters of 6 and 8 mm were used for mixing. The balls were selected to coat the WC powder grains with ductile cobalt best. The mixing process was carried out in two stages. The first stage consisted of the preparation of WC powder (94 wt.%) + Co (6 wt.%) for the composite matrix, while the second step was to prepare the final blend of WC-Co with diamond (30 vol.%).

**Figure 3:**
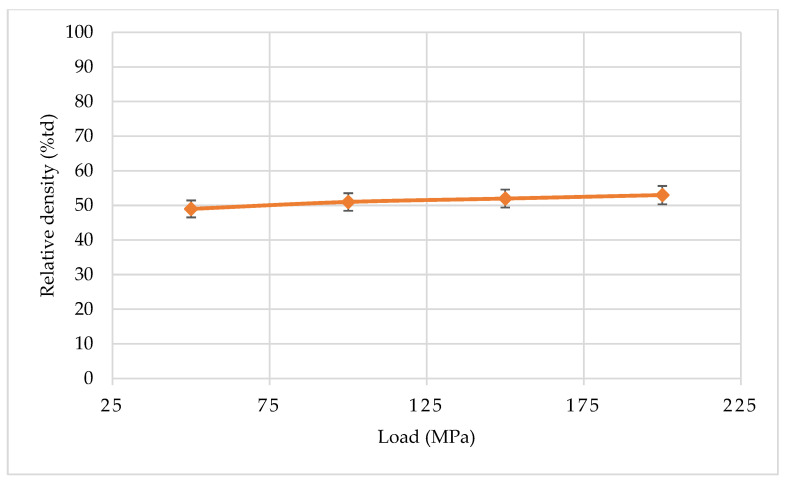
Impact of pressing pressure on density of WC-Co.

To study the effect of precompaction on the density of the resulting moldings, a hydraulic press in the pressure range of 50–200 MPa was used. Figure 3 presents the dependence of the relative density of molded parts on pressing pressure.

Based on the tests carried out, it can be concluded that precompaction has little impact on the final density of the molded part. Samples compacted at 50 MPa have a density of about 49% of the theoretical density, while those compacted at 200 MPa have density of about 53%. Thus, the increase in density is small—about 4%. Therefore, initial compaction of 50 MPa was assumed for all sintering processes. The process of powder densification was performed in a graphite matrix immediately before the sintering process.

**Figure 4:**
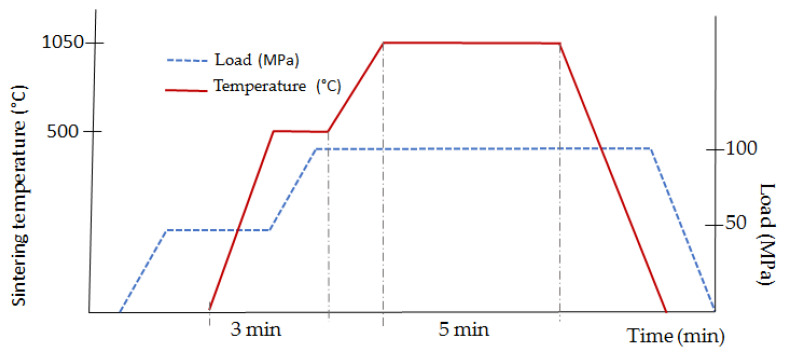
Schematic course of the consolidation process of the WC-Co/diamond composite.

The results of the research presented in the paper [32] showed that in order to obtain a solid WC-Co composite, it is enough to carry out the sintering process at a temperature of 1050 °C, under a load of 100 MPa. Therefore, the sintering process of WC-Co composites with diamond was performed in two stages. The degassing stage was carried out at a temperature of about 600 °C for 3 min, increasing the load from 50 to 100 MPa in the second minute. In the second stage, the sintering itself was carried out at 1050 °C for 5 min (Figure 4).

## 3. Results

**Figure 5:**
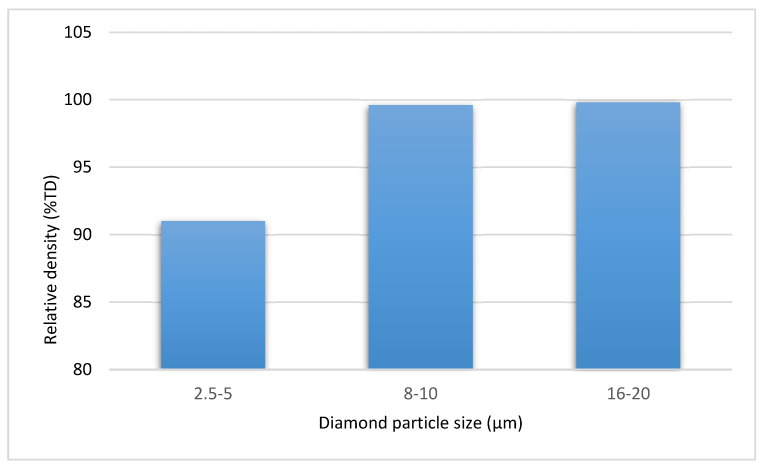
Impact of the diamond size on the density of the WC-Co/diamond composites.

The lowest relative density—91%—was characteristic for composites with the smallest diamond particles, 2.5–5 μm in size. The composites consolidated with the use of larger diamond gradations: 8–10 and 16–20 μm had much higher relative density (above 99% of the theoretical density) (Figure 5).

**Figure 6:**
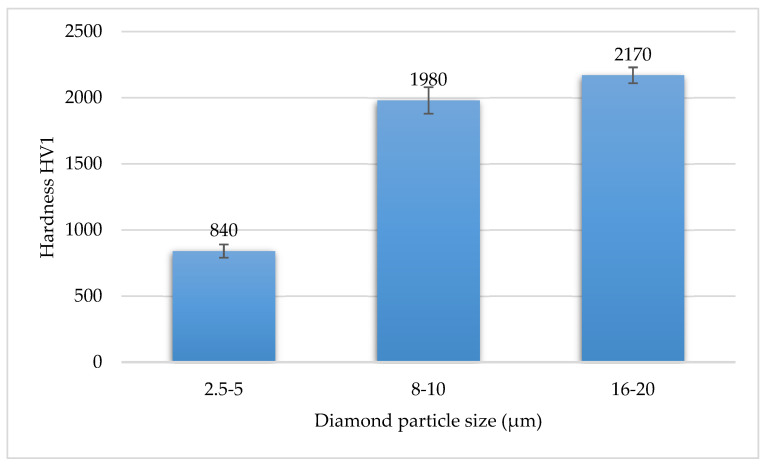
Impact of diamond particle size on the hardness of WC-Co/diamond composite.

As the diamond particle size increased, the hardness of the WC-Co/diamond composites increased as well. Sinters with diamond particles of 2.5–5 μm had a very low hardness of about 840 HV1. Sinters in which the largest diamond particles of 16–20 μm gradation were used had the highest hardness—2170 HV1 (Figure 6). The hardness of composites containing 16–20 µm diamond particles is higher compared to WC-Co sinters obtained by the PPS method [32].

**Figure 7:**
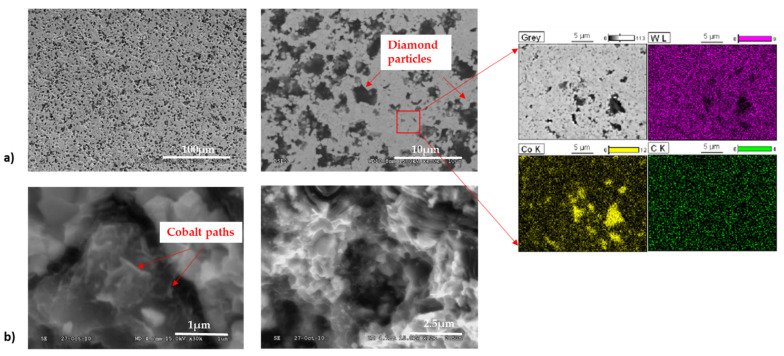
SEM images of WC-Co/diamond 2.5–5 μm composite: (**a**) surface microstructure end EDS results, (**b**) fracture microstructure.

Microstructure images of the fracture and surface of composites containing the 2.5–5 μm diamond revealed the presence of graphite (Figure 7). The surface of the diamond is strongly developed and degraded. No transcrystalline fractures through the diamond particles were observed.

**Figure 8:**
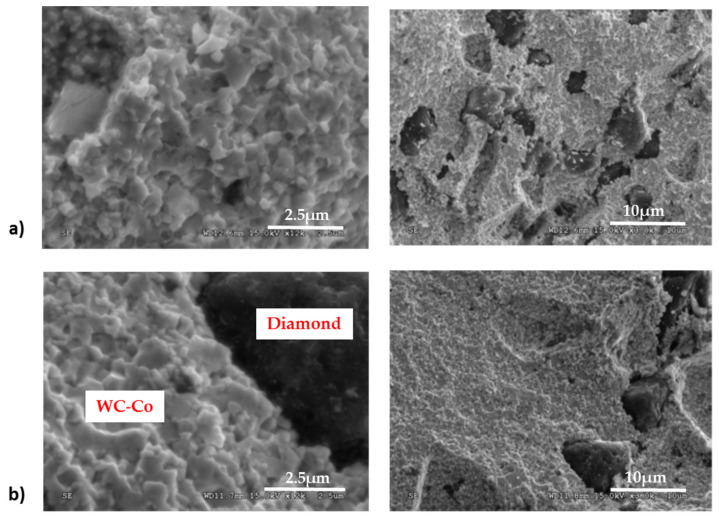
SEM images of WC-Co/diamond composites (**a**) 8–10 μm, (**b**) 16–20 μm.

Composites containing diamond particles: 8–10 and 16–20 μm are characterized by well-anchored diamond particles in the matrix (Figure 8). Images of the fracture microstructure show numerous transcrystalline breaks through the diamond particles, indicating good bonding to the matrix. The matrix of the composite is characterized by well-formed WC grains with characteristic sharp edges. The cobalt is distributed evenly across the WC grain boundaries.

The low density and hardness of composites with diamond particles of 2.5–5 μm are related to the presence of graphite, which is also confirmed by the analysis of phase composition carried out by X-ray diffraction method. XRD recording in a narrow angular range, including the presence of graphite, was performed to verify the presence of this phase.

Despite the use of identical sintering conditions, the diamond particles were significantly degraded—transformed into graphite. The activation energy of the transformation of diamond into graphite depends on the internal and external structure of the crystal. Studies have shown that the rate of diamond graphitization is influenced not only by temperature but also by the size of the diamond particles [17,18,19,20,21,22,23].

**Figure 9:**
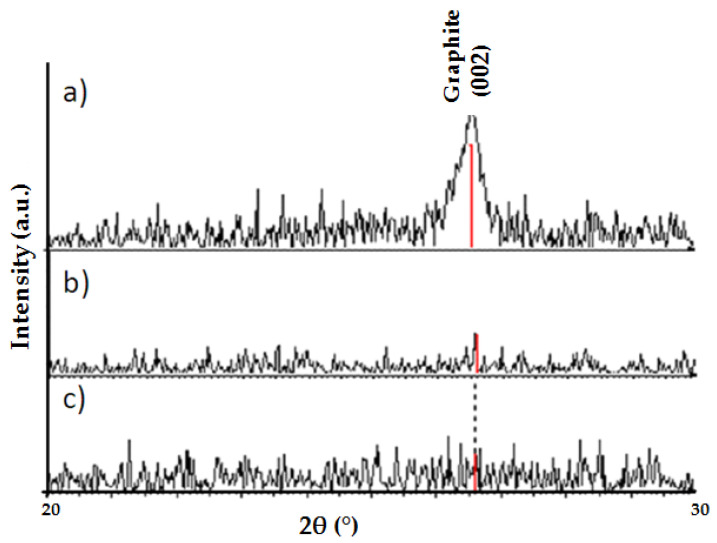
Diffraction patterns of WC-Co/diamond composites with different diamond particle sizes: (**a**) 2.5–5 μm, (**b**) 8–10 μm, (**c**) 16–20 μm.

In sinters containing diamonds of particle size 8–10 and 16–20 μm, the presence of graphite was not detected by the method used (Figure 9).

**Figure 10:**
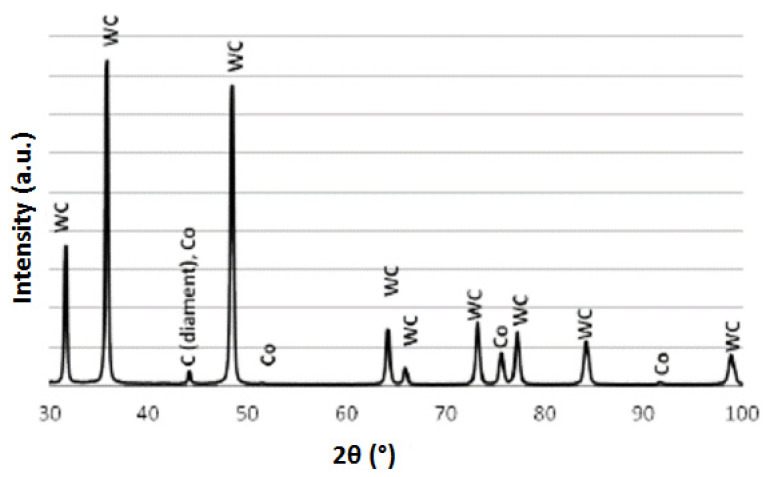
Diffraction analysis of WCCo/diamond composite (8–10 μm and 16–20 μm).

Full diffraction record of the WC-Co composite with 8–10 μm and 16–20 μm diamond, presented in Figure 10, allows the identification of three phases: WC, Co and C (diamond).

**Figure 11:**
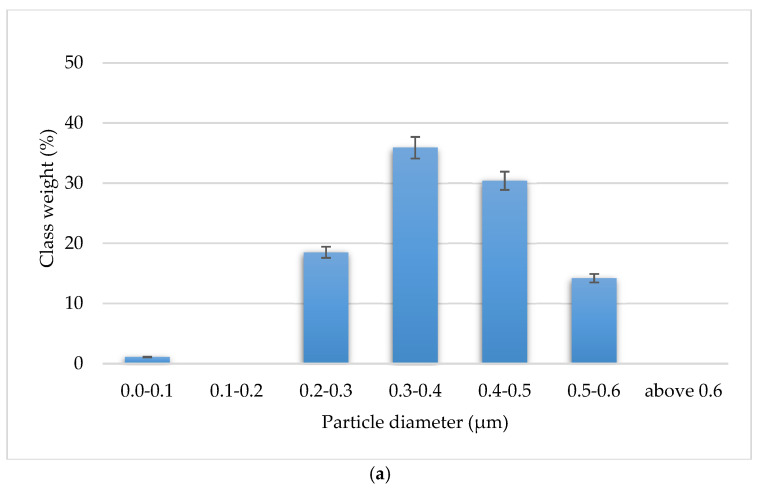

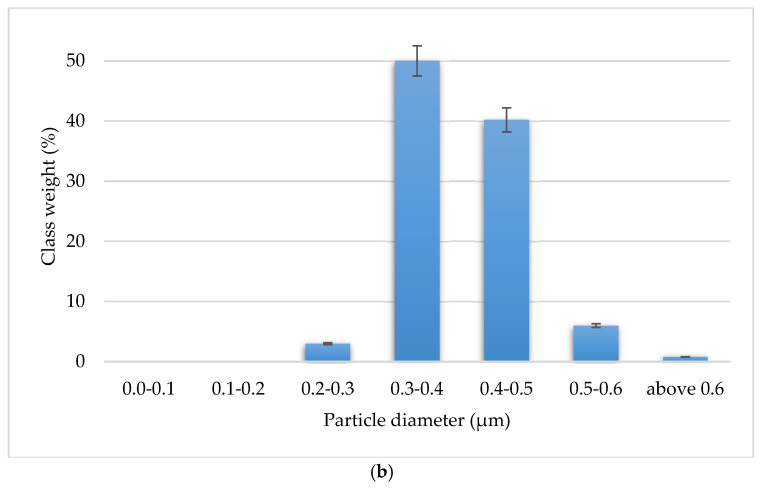
WC grain size distribution: (**a**) in WC-Co/diamond composites, (**b**) in WC starting powder.

The WC grain sizes of the obtained composites were analyzed with the particle size of the WC powder used to obtain the sinter. The average WC grain size in the solid composites was 0.4 ± 0.13 μm and is the same as the grain size of the starting powder. Thus, no WC grain growth occurred during the sintering process. Figure 11 presents histograms showing the distribution of WC grain size in the sintered composites and the starting powder.

## 4. Summary

In the formation of the WC-Co/diamond composites, the main problem is the graphite removal from the diamond–matrix contact zone. At present in solving this problem the best results have been attained by doping the composite with carbide forming chemical elements [33,34]. The authors in [25] presented a comparison of the results of WC-Co composites with a diamond-coated and not covered with a tungsten layer. The coating was applied by vapor deposition in a vacuum. The composites were sintered by the SPS method. Studies have shown that in a composite sintered with uncoated diamond particles, at a temperature of 1000 °C, the diamond is only mechanically bonded to the matrix. There are gaps between the diamond particle and the matrix, and graphite precipitation is visible at the diamond-matrix interface, which of course significantly deteriorates the properties of the sinter. Only in composites containing diamond particles coated with tungsten, the presence of graphite is not disclosed. Thus, it is not possible to obtain a composite of tungsten carbide with dispersed diamond particles using the SPS technology (without the use of a coating on the diamond particles).

The conventional sintering process favors the growth of WC grains. It is carried out in two stages. In the pre-sintering phase, diffusion of C and WC into γ-Co occurs in the temperature range of 600–1250 °C, and then at higher temperatures.

Cobalt melts and some of the tungsten carbide WC is dissolved in it. During cooling, the dissolved WC is released from the γ phase, which contributes to grain growth.

The PPS method proceeds differently from conventional sintering due to different heating conditions. A local, significant increase in the powder temperature is forced by the current flow, and after the current disappears, the powder cools down intensively to the preset sintering temperature. This is accompanied by an intensive transport of material into the area of the formed necks. A uniform distribution of cobalt is observed at the WC grain boundaries, which indicates that consolidation in the PPS method also takes place in the presence of the liquid cobalt phase. If the process was carried out without the participation of the liquid phase, the cobalt would appear in the form of agglomerates. In the PPS sintering method, the short duration of high temperature and its rapid decrease strongly limit grain growth.

The XRD analysis showed the occurrence of Co in addition to Co in the high-temperature variety with an increased lattice parameter (Coreg). This indicates that the dissolution of carbon and tungsten in cobalt occurs during the sintering process. These elements have an impact on the stabilization of the high-temperature variety of cobalt [12,33].

## 5. Conclusions

The study of WC-Co/diamond composites carried out showed that by sintering at 1050 °C under a load of 100 MPa a solid composite with 8–10 and 16–20 μm diamond particles can be obtained. The applied method, however, does not allow to obtain a composite with smaller diamond particles, from the range of 2.5–5 μm. These sinters have low density and the diamond particles are so small that they undergo graphitization under the applied sintering conditions easily and quickly. The sintering conditions applied are not optimal for obtaining a solid composite containing 30% diamond, size 2.5–5 μm.

## Data Availability

Data sharing is not applicable to this article.
